# Phase I Trial of Fourth-Generation Anti-CD19 Chimeric Antigen Receptor T Cells Against Relapsed or Refractory B Cell Non-Hodgkin Lymphomas

**DOI:** 10.3389/fimmu.2020.564099

**Published:** 2020-11-27

**Authors:** Xuan Zhou, Sanfang Tu, Chunsheng Wang, Rui Huang, Lan Deng, Chaoyang Song, Chunyan Yue, Yanjie He, Jilong Yang, Zhao Liang, Anqin Wu, Meifang Li, Weijun Zhou, Jingwen Du, Zhenling Guo, Yongqian Li, Cheng Jiao, Yuchen Liu, Lung-Ji Chang, Yuhua Li

**Affiliations:** ^1^ Department of Hematology, Zhujiang Hospital, Southern Medical University, Guangzhou, China; ^2^ Department of Hematology, Shantou Central Hospital, Shantou, China; ^3^ Department of Research and Development, Geno-Immue Medical Institute, Shenzhen, China; ^4^ School of Medicine, University of Electronic Science and Technology of China, Chengdu, China; ^5^ Bioland Laboratory (Guangzhou Regenerative Medicine and Health Guangdong Laboratory), Guangzhou, China

**Keywords:** CD19, chimeric antigen receptor T cells, non-Hodgkin’s lymphomas, cytokine release syndrome, B cell lymphoma

## Abstract

**Background:**

The administration of second- or third-generation anti-CD19 chimeric antigen receptor (CAR) T cells has remarkably improved the survival of patients with relapsed or refractory B cell malignancies. However, there are limited clinical results from fourth-generation CAR-T cell therapy, and the factors affecting response rate and survival have not been fully determined.

**Methods:**

Lymphoma patients with progression or relapse after intensive treatments, including hematopoietic stem cell transplantation, and life expectancy >2 months were enrolled in the study. Peripheral lymphocytes were collected through apheresis, and magnetically selected T cells were lentivirally transduced with a 4th-generation CAR featuring an anti-CD19 CAR and the iCasp9 suicide switch (4SCAR19). The patients received 4SCAR19 T cell infusion after approximately seven days of expansion and a conditioning regimen comprising cyclophosphamide/fludarabine. The efficacy, safety, and risk factors were evaluated.

**Results:**

A total of 21 patients with relapsed/refractory B cell non-Hodgkin lymphoma were enrolled and received 4SCAR19 T cell infusions at a median dose of 8.9×10^5^ CAR-T cells/kg. The overall response rate was 67% [95% confidence interval (CI), 43 to 85], with 43% of patients achieving a complete response and 24% having a partial response. The overall and complete response rates were 58 and 33% in the diffuse large B-cell lymphoma (DLBCL) group and 78 and 56% in the non-DLBCL group, respectively. The median overall survival was 23.8 months (95% CI, not reached), with a median follow-up of 13.7 months. Factors affecting overall survival were International Prognostic Index (IPI), disease type, and remission status after CAR-T cell treatment. The most common adverse events of grade 3 or 4 during treatment were neutropenia (76%), leukopenia (71%), and thrombocytopenia (29%). The incidence of cytokine release syndrome (CRS) was 14%, and all cases were grade 1. One patient developed grade 3 neurotoxicity. No deaths were attributed to infusion of 4SCAR19 T cells, CRS, or neurotoxicity.

**Conclusions:**

In this study, patients with relapsed or refractory B cell non-Hodgkin’s lymphoma who received 4SCAR19 T cell therapy had durable responses and few of adverse events. The IPI model is suitable for evaluating the prognosis of patients receiving CAR-T cell therapy.

**Trial registration:**

Chinese Clinical Trial Registry (http://www.chictr.org.cn): ChiCTR-OOC-16007779.

## Introduction

B cell non-Hodgkin’s lymphomas (NHLs) are the most common subtype of NHLs, accounting for 86.6% of NHLs in developing countries and 90.7% of NHLs in developed nations (1, 2). Recent years have seen a significant improvement in the overall survival of patients with NHL; the 5-year survival rate of patients receiving first-line treatment is between 50 and 70% (3, 4). However, 30% to 50% of patients are refractory to the standard treatment or relapse after remission (4, 5). The prognosis of these patients is extremely poor, with a dismal objective response rate of 26% and a median overall survival of 6.3 months after salvage treatment, as shown in SCHOLAR-1 research (5), highlighting the need for alternative therapies in patients with refractory/relapsed (R/R) lymphoma.

Anti-CD19 chimeric antigen receptor (CAR) T cells have emerged as a promising approach to treat B cell malignancies and showed an objective response rate of 52~83% in the pivotal trials JULIET and ZUMA-1 (6–8), resulting in their approval by the Food and Drug Administration (FDA) for patients with NHL. In addition, axicabtagene ciloleucel exhibited a slightly lower objective response rate of 59~64% in the real world than in ZUMA-1 (9, 10). Despite the remarkable efficacy of CAR-T cell therapy, another issue that cannot be neglected is the concurrent toxicity due to genetically modified T cells. The two most common life-threatening adverse reactions are cytokine release syndrome (CRS) and neurotoxicity (also known as CAR-T-cell-related encephalopathy syndrome, CRES). According to reports, the rate of CRS associated with CAR-T cell immunotherapy in lymphoma was as high as 58 to 96%, while the CRES incidence rate was 21 to 76% (6–10).

A novel strategy of inserting suicide genes into transduced T cells has been developed to address this issue. The most frequently used suicide gene is inducible caspase 9 (iCasp9), which is modified and fused with an FK506 binding protein variant (11–13). After induction by chemical inducers of dimerization (CIDs), 99% of T cells with high transgene expression undergo apoptosis without loss of normal cells *in vitro* and *in vivo* (11). The iCasp9 suicide switch was transduced into donor T cells for haploidentical stem cell transplantation in a phase I clinical trial, and administration of CIDs effectively terminated GVHD without relapse (14). In addition, preclinical studies combining the iCasp9 gene with anti-CD20 or anti-CD19 CAR-T cells have proven the feasibility and prospects of this suicide switch (15–17). However, the data regarding the clinical application of iCasp9 in CAR-T cell platforms are limited.

Moreover, no differences in objective response between prognostic subgroups were found in the JULIET trial, while ongoing responses were reported to be correlated with CAR-T cell expansion in ZUMA-1. However, the factors affecting survival have not been fully determined. Here, we report the effectiveness and safety of our clinical study using 4th-generation CAR-T cells featuring an anti-CD19 CAR and the iCasp9 gene in patients with R/R B cell NHL and evaluated the risk factors affecting response rate and survival.

## Methods

### Study Design and Participants

We performed a single-arm, open-label, multicenter, phase I study enrolling patients from four clinical centers. Eligible participants had to be at least 18 years old and had B cell lymphoma in the refractory/relapsed status, which was defined as not reaching complete remission after four cycles of chemotherapy, including rituximab at the time of initial treatment, or two cycles of salvage treatment or having disease progression or relapse after complete remission with immunochemotherapy or hematopoietic stem cell transplantation (HSCT). Patients confirmed to be CD19 positive through immunohistochemistry or flow cytometry were included. Patients with both invasive and indolent B cell lymphoma were included in this trial, including but not limited to DLBCL, follicular lymphoma (FL), primary mediastinal large B cell lymphoma (PMBCL), and double- or triple-hit lymphoma (DHL/THL) with MYC, BCL2, and/or BCL6 rearrangements. Patients who had formerly received any gene therapy or cell therapy, those who had uncontrolled acute life-threatening infections, or those exposed to graft-*versus*-host disease (GVHD) requiring immunosuppressive agents were excluded. Those who used systemic steroids and immunosuppressants within 2 weeks prior to treatment were excluded.

The clinical trial followed the guidelines of the Declaration of Helsinki and was approved by the institutional review board of Zhujiang Hospital of Southern Medical University. The trial has been registered in the Chinese Clinical Trial Registry (number, ChiCTR-OOC-16007779). Written informed consent was obtained before screening.

### Manufacture and Infusion

The individualized CAR-T cells were produced as previously described (18). Briefly, peripheral blood mononuclear cells of eligible participants were obtained through apheresis and transported to Geno-Immune Medical Institute, Shenzhen, China, where the CAR-T product was manufactured. CD3-positive T cells were selected and activated using CD3/CD28 magnetic beads. Then, the T cells were lentivirally transduced with a fourth-generation anti-CD19 CAR containing various costimulatory signaling domains we synthesized a codonoptimized CD19 CAR (19z) based on anti-CD19 scFv of the mouse hybridoma FMC63 (19), and constructed a fourth generation 19z CAR, which contained a chimeric intracellular signaling element incorporating CD28 trans-membrane and cytoplasmic domain, the cytoplasmic domains of CD27 (273z, amino acid 213–260), and the CD3ζ chain intracellular domain with an inducible caspase 9 gene as previously described (20, 21). The detail of the full sequence was covered in the patent (WO2019161796 A1). After 5–7 days of expansion, 4SCAR19 T cells were harvested and transported back to the medical institution for reinfusion.

**Figure 1:**
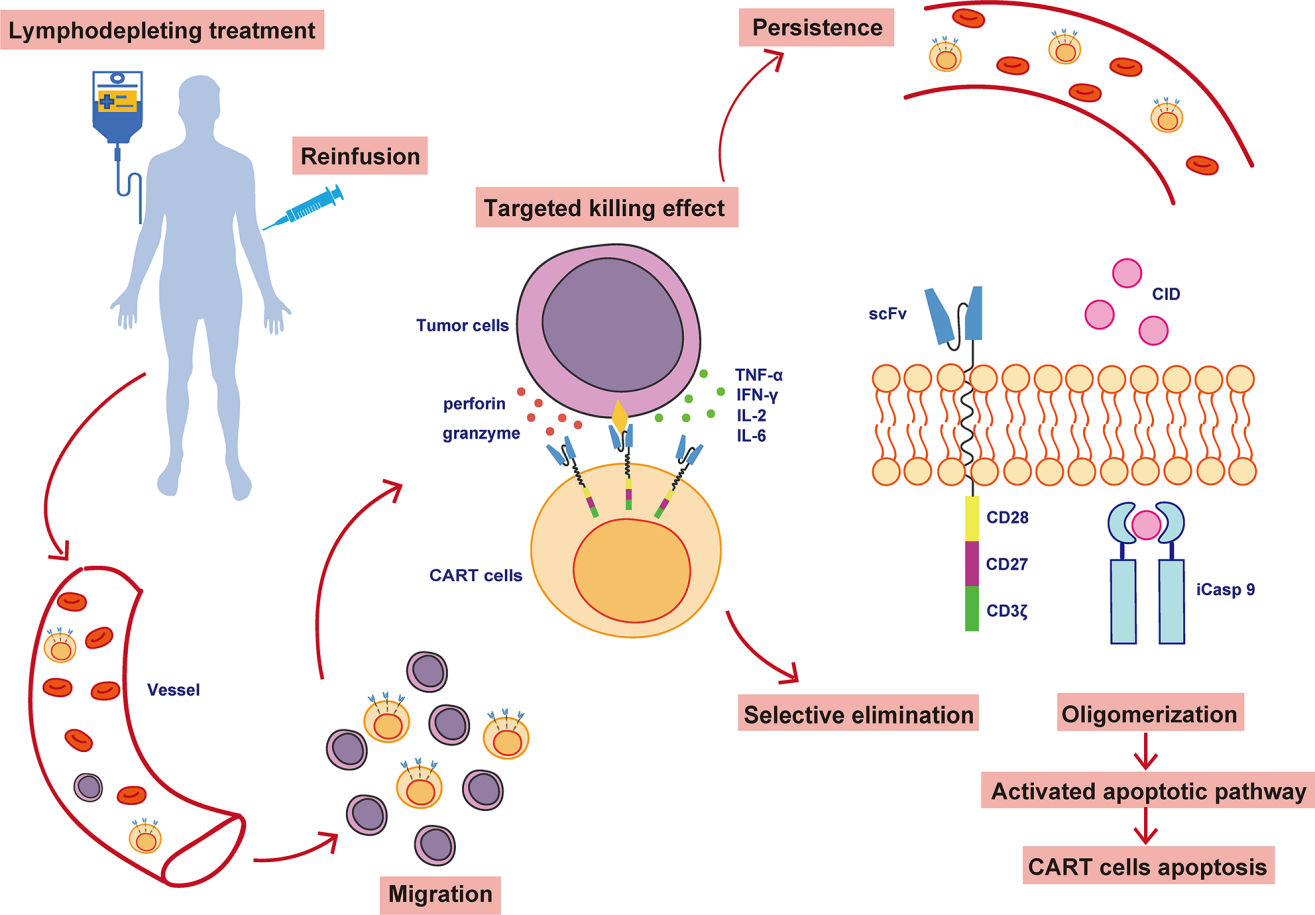
The structure and *in vivo* activity of 4SCAR19 T cells. CAR-T cells, chimeric antigen receptor T cells; scFv, single-chain variable fragment; iCasp 9, inducible caspase 9; CID, chemical inducer of dimerization.

Participants received a 3-day conditioning regimen (fludarabine, 25 mg/m^2^ for 3 days; cyclophosphamide 900 mg/m^2^ for one day) to clear circulating endogenous lymphocytes and immunosuppressive cells. Some patients with a high tumor burden also underwent bridging chemotherapy based on previous chemotherapy regimens to decrease the probability and severity of CRS. Subjects were infused with 4SCAR19 T cells on day 0, and their clinical manifestations and serum marker levels were closely monitored. The structure of the 4SCAR19 construct and its *in vivo* activity are shown in **Figure 1**. Participants were continuously monitored clinical responses in a preset timeline. The CAR copy number in the blood was determined by quantitative real-time polymerase chain reaction (qPCR; based on both SYBR and TaqMan probe methods) and the cytokine analysis based on cytokine bead array (CBA) as previously described (21).

### End Points

Efficacy was evaluated according to the standards recommended by the International Working Group (IWG) in 1999, and the first evaluation was performed 30 days after CAR-T cell infusion. The primary endpoint was the overall response rate (ORR), defined as the proportion of patients who achieved remission. Secondary end points included duration of response (DoR), event-free survival (EFS), overall survival (OS), and incidence and severity of adverse events. Safety was evaluated according to the CTCAE V4.03 standard, CRS was graded according to a grading system known as the Lee criteria (NCI), and CAR-T-cell-related encephalopathy syndrome (CRES) was scored according to the MDACC CRES grading system (22, 23).

### Statistical Analysis

The 95% confidence interval (CI) of the response rate was calculated using the Clopper-Pearson method. The overall response rate of subgroups was compared by chi-square test and Fisher’s exact probability method. The differences between groups were compared by one-way ANOVA. Univariate analysis was performed by the log-rank test. Survival rates were estimated with the Kaplan–Meier method and compared by applying the log-rank test. A two-sided P value<0.05 was considered statistically significant. Analyses were performed with SPSS, version 20.

## Results

### Characteristic of Patients

Between May 2016 and the data cutoff date, September 17, 2019, a total of 21 patients aged 31 to 77 years old with R/R NHL were enrolled and received an infusion with CD19 CAR-T cells. Eighteen (86%) patients had aggressive B cell lymphoma, and the other three patients had indolent lymphoma. Of the 12 patients with DLBCL, 1 patient had central nervous system DLBCL. Most of the patients had been heavily pretreated, with five (24%) undergoing more than five lines of antitumor chemotherapy and five patients undergoing autologous HSCT. However, the patients still had a heavy tumor burden; the longest diameter of the affected lymph nodes of 5 patients was ≥ 5 cm, and 13 patients had more than 5 involved lymph nodes. Seventeen out of 21 patients (81%) had extranodal lesions, and the most common sites for extranodal lesions were spleen (17%), gastrointestinal tract (14%), bone (14%), pleura (14%), and lungs (11%). Fourteen patients had refractory B-NHL (patients who failed to respond to the last therapy), and seven patients had relapsed B-NHL (patients who had a partial or complete response after the last line of therapy but relapsed before enrollment in this trial). The baseline characteristics of the enrolled patients are summarized in **Table 1**.

**Table 1:** Baseline demographics and clinical characteristics.

Characteristic	Patients (%)*
Gender	
Male	13 (62)
Female	8 (38)
Age (year)	
<60	11 (52)
≥60	10 (48)
ECOG performance status	
0 ~ 1	19 (90)
2 ~ 4	2 (10)
Diagnosis at screening	
Diffuse large B-cell lymphoma	12 (57)
Mantle cell lymphoma	3 (14)
High-grade B-cell lymphoma (double/triple-hit)	1 (5)
Follicular lymphoma	2 (10)
Burkitt lymphoma	1 (5)
Gastric MALT lymphoma	1 (5)
Primary mediastinal large B-cell lymphoma	1 (5)
Disease stage at screening	
Stage I	0
Stage II	1 (5)
Stage III	5 (24)
Stage IV	15 (71)
No. of previous lines of antineoplastic therapy^§^	
1 ~ 5	16 (76)
6 ~ 10	5 (24)
Longest diameter of lymph node (cm)	
<5	11 (52)
≥5	5 (24)
Unknown	5 (24)
Number of extranodal lesions	
0	4 (19)
1 ~ 2	12 (57)
3 ~ 4	5 (24)
Disease status	7 (33)
Refractory disease	14 (67)
Relapsed disease	7 (33)

*The total percentage may not be 100 because of rounding.

^§^Therapies containing rituximab and anthracycline or hematopoietic stem cell transplantation were counted. For those who had transformed lymphoma, the lines of antineoplastic therapy were counted after transformation.

ECOG, Eastern Cooperative Oncology Group.

In the previous study of our center, it was found that compared with patients with low tumor burden, leukemia patients with higher tumor burden are less likely to achieve remission after 4SCAR19 infusion, and are accompanied by a higher probability of relapse/progression (18). Therefore, take the prognosis of patients into consideration, we administered bridging chemotherapy to seven patients with high tumor burden in this trial. Of all enrolled patients, 86% (18/21) received a conditioning regimen of combination fludarabine-cyclophosphamide, one patient received rituximab and fludarabine-cyclophosphamide, and the other two patients received fludarabine or cyclophosphamide alone. All 21 patients received a median dose of 8.9×10^5^/kg 4SCAR19-positive viable T cells (range, 0.3×10^5^/kg to 48.0×10^5^/kg) (**Supplemental Table S1**). For all patients, the median time from leukapheresis to CAR-T cell infusion was 16 days.

### Efficacy

**Figure 2:**
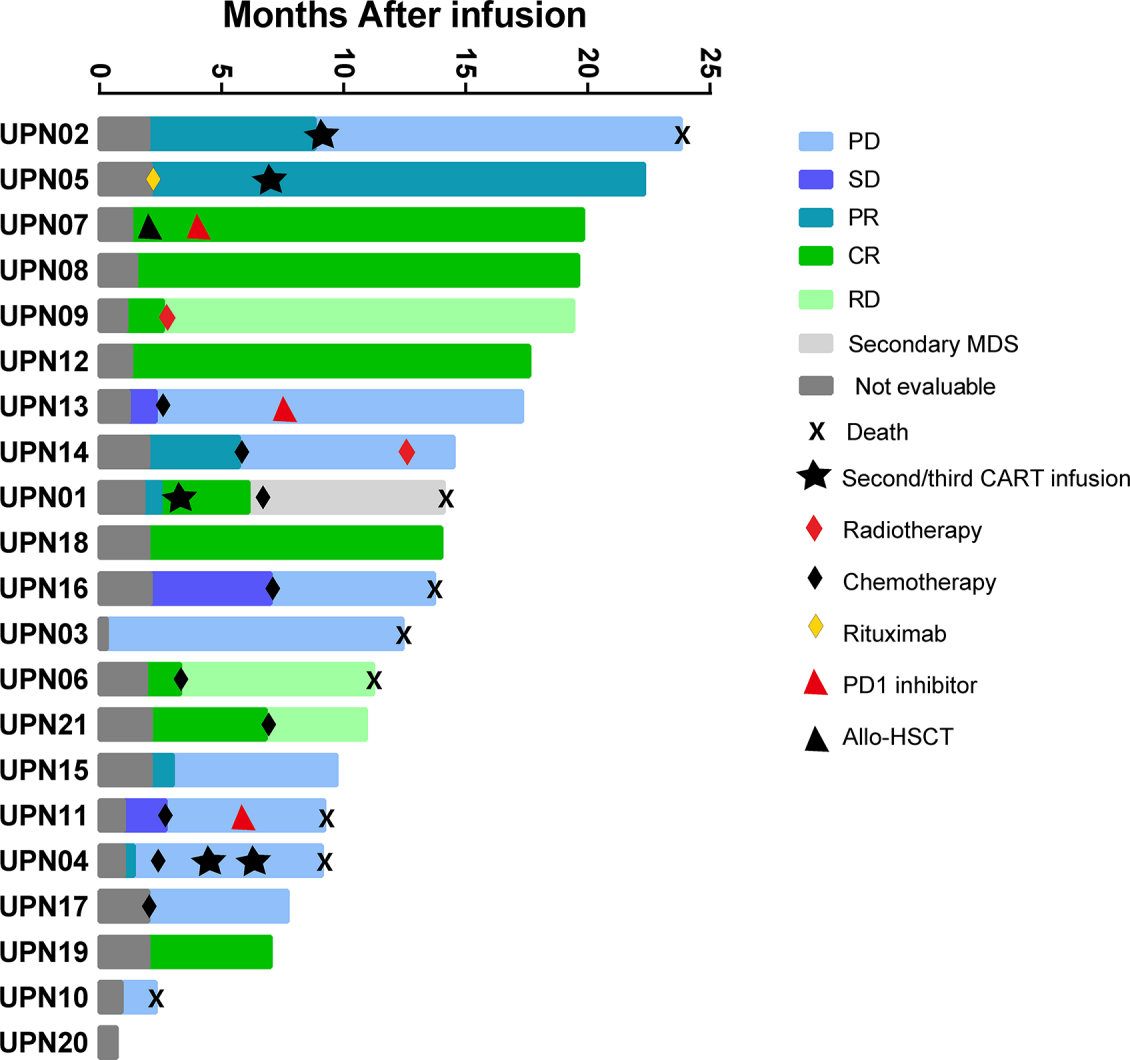
The waterfall plot of individual response. Allo-HSCT, allogeneic hematopoietic stem cell transplantation; CR, complete response; MDS, myelodysplastic syndrome; PD, progressive disease; PR, partial response; RD, relapsed disease; SD, stable disease.

**Figure 3:**
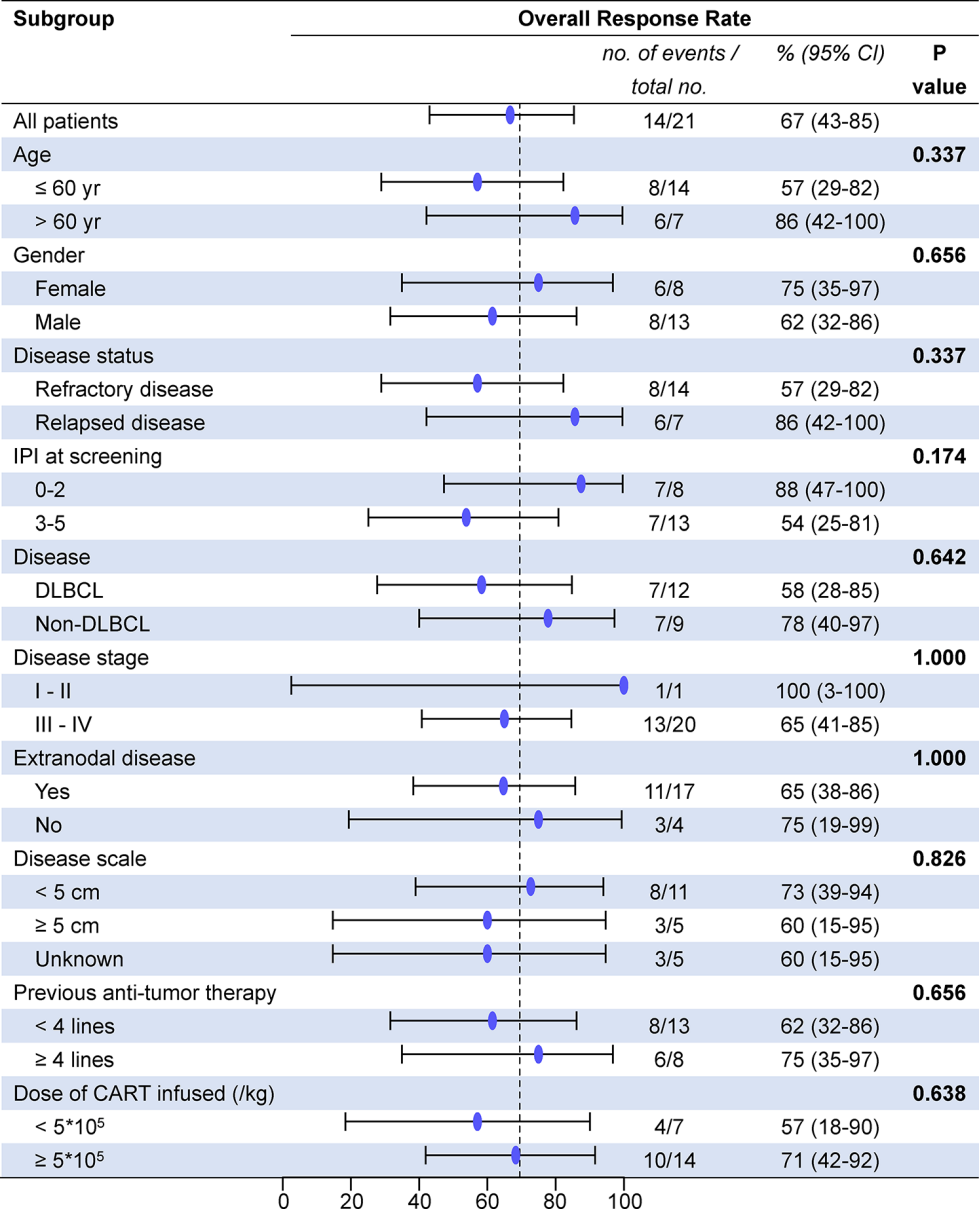
Overall response rate between subgroups. DLBCL, diffuse large B cell lymphoma; IPI, International Prognostic Index; CAR-T cells, chimeric antigen receptor T cells; CI, confidence interval.

All patients who received 4SCAR19 T cell infusion were included in the efficacy analysis, and the median follow-up was 13.7 months (range, 0.7 to 23.8) in the cohort. The best ORR was 67% (95% CI, 43 to 85), with 43% of patients achieving a complete response and 24% having a partial response. The rates of overall and complete response at month 3 were 48 and 33%, respectively, and 43 and 33% at month 6. The rates of overall and complete response were 58 and 33%, respectively, in the DLBCL group and 78 and 56% in the non-DLBCL group. Individual response is shown in the waterfall plot (**Figure 2**). Among seven patients who received debulking therapy before conditioning, two of them achieved partial response and five of them achieved no response to debulking. One of seven patients achieved complete response, three patients achieved partial response, one patient had stable disease, and the other two patients faced progression after CAR-T cell infusion. In the subgroup analyses, there was no notable difference in the overall response rate in the demographic and prognostic subgroups (**Figure 3**). Besides, T cell phenotypes were assessed with the CD4+ and CD8+ T cells re-infused at a ratio approximately at 1:1, and no correlation was found between T cell phenotypes and clinical responses.

**Figure 4:**
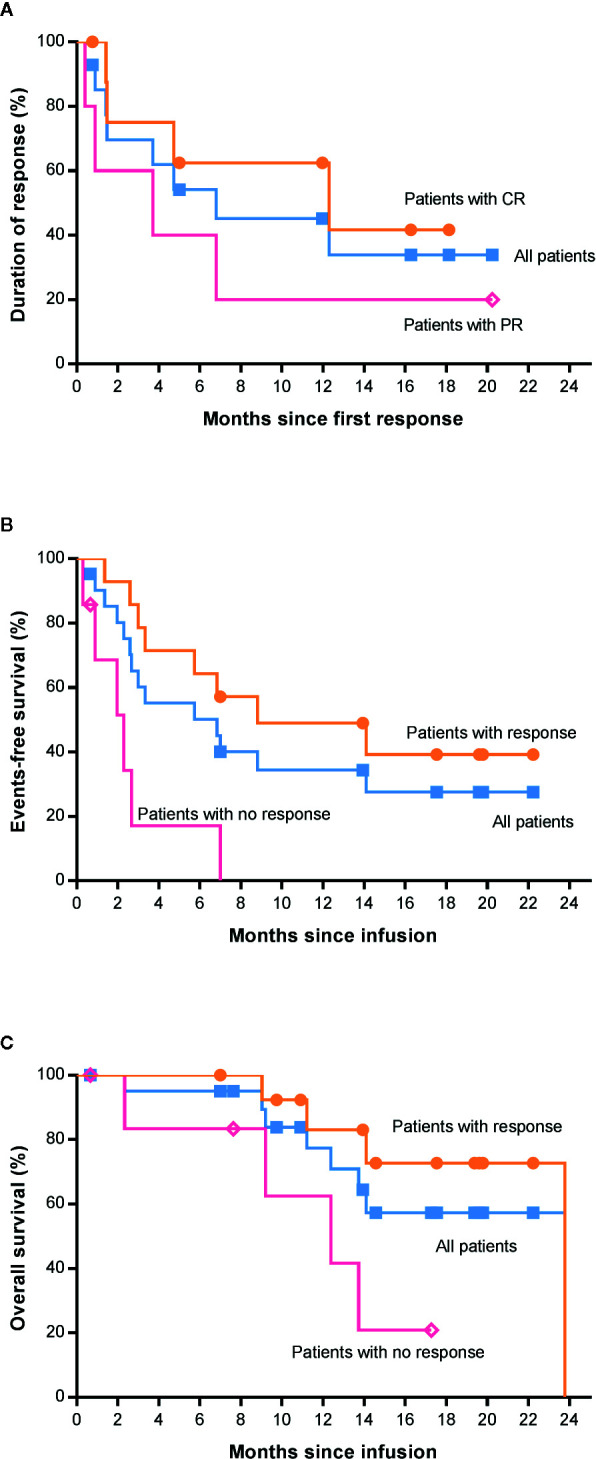
Survival analysis of patients by the Kaplan-Meier method. **(A)** Duration of response among the 14 patients who had a response. **(B)** Event-free survival between patients with and without response. **(C)** Overall survival between patients with and without response. CR, complete response; PR, partial response; HSCT, hematopoietic stem cell transplantation.

The median time to first documented remission post infusion in all patients who achieved remission was 58 days (range, 29 to 63). There was no significant difference in time to remission after infusion in patients with complete remission or partial remission (P=0.509, **Supplemental Table S2**). The median DoR of patients with CR or all patients who achieved a response was 12.3 and 6.8 months, respectively, while it was 3.7 months in patients with PR. It is expected that 63% (95% CI, 23 to 86) of patients with CR and 45% (95% CI, 18 to 69) of all patients will remain relapse-free or progression-free at 12 months after remission (**Figure 4A**). Until the data cutoff date, a sustained remission of up to 20.2 months was observed after infusion, and five patients remained in remission for 5 to 20 months. After 0.4–6.8 months of remission, seven patients developed relapse or progression, but no CD19-negative relapse was found. The B cell count in these patients was close to zero at infusion time, and normal B cells did not rebound when relapsed. One patient underwent HSCT 23 days after confirmation of CR by positron emission tomography/computed tomography (PET-CT) after infusion and maintained sustained remission. Six patients who did not respond received chemotherapies and/or radiotherapy, and none of them proceeded to HSCT.

The median EFS was 6.8 months in all patients (**Figure 4B**). In addition, we found that there was a significant difference in EFS between patients with and without a response (P=0.0014, **Figure 4B**), and the median EFS in patients with response was longer than that in those with no response (8.8 *vs.* 2.3 months). The median OS was 23.8 months (95% CI, not reached) among all 21 patients (**Figure 4C**). However, the median OS among patients who had a complete response has not been reached. The estimated rate of OS at 12 months after 4SCAR19 T cell infusion was 77% (95% CI, 50 to 91) and 86% (95% CI, 33 to 98) in all patients and patients with complete response, respectively. It is estimated that 57% (95% CI, 30 to 77) of all patients who received an infusion will survive at 22 months. As of the data cutoff date, 7 of 21 patients died of relapse or disease progression, and 1 patient died of a secondary tumor.

In the univariate analysis of OS, we found that International Prognostic Index (IPI) score, disease subtype, and response status after CAR-T cell infusion were related to the survival time of patients (**Table 2**). The IPI score was proposed by Shi PP *et al*. in 1993 to assess the prognosis of NHL, and in the IPI, a higher score indicates a worse prognosis. In this trial, patients with low risk and low-medium risk survived longer than patients with intermediate risk and high risk (p=0.031, HR=7.742), suggesting that the IPI model is suitable for assessing the prognosis of patients who received CAR-T cell therapy. This analysis also showed that patients who achieved response after CAR-T cell treatment had a lower risk of death than those without response (P=0.025, HR=4.855, **Figure 4C**).

**Table 2:** Univariate analysis of overall survival time.

Subgroup	No. of patients	χ^2^	P value	HR (95% CI)
Age				
≤60 year	14	2.802	0.094	
>60 year	7			
Gender				
Female	8	1.193	0.275	
Male	13			
Disease status				
Refractory disease	14	0.152	0.697	
Relapsed disease	7			
IPI at screening				
0–2	8	4.639	0.031	**1.000**
3–5	13			**7.472 (0.885–63.067)**
Disease subtype				
DLBCL	12	5.322	0.021	**1.000**
Non-DLBCL	9			**0.116 (0.013–1.000)**
Disease stage				
I–II	1	0.581	0.446	
III–IV	20			
Extranodal disease				
Yes	17	0.152	0.696	
No	4			
Disease scale				
<5 cm	11	1.205	0.547	
≥5 cm	5			
Unknown	5			
Previous therapies				
<4 lines	13	0.166	0.684	
≥4 lines	8			
Dose of CAR-T cells				
<5*10^5^ **/**kg	7	1.109	0.292	
≥5*10^5^ **/**kg	14			
Response status				
Response	14	5.007	0.025	**1.000**
No-response	7			**4.855 (1.059–22.248)**

*The total percentage may not be 100 because of rounding.

^§^Bolded value in table 2 was to emphasize HR (Hazard Ratio) between the exposure group and the non-exposed group.

**Figure 5:**
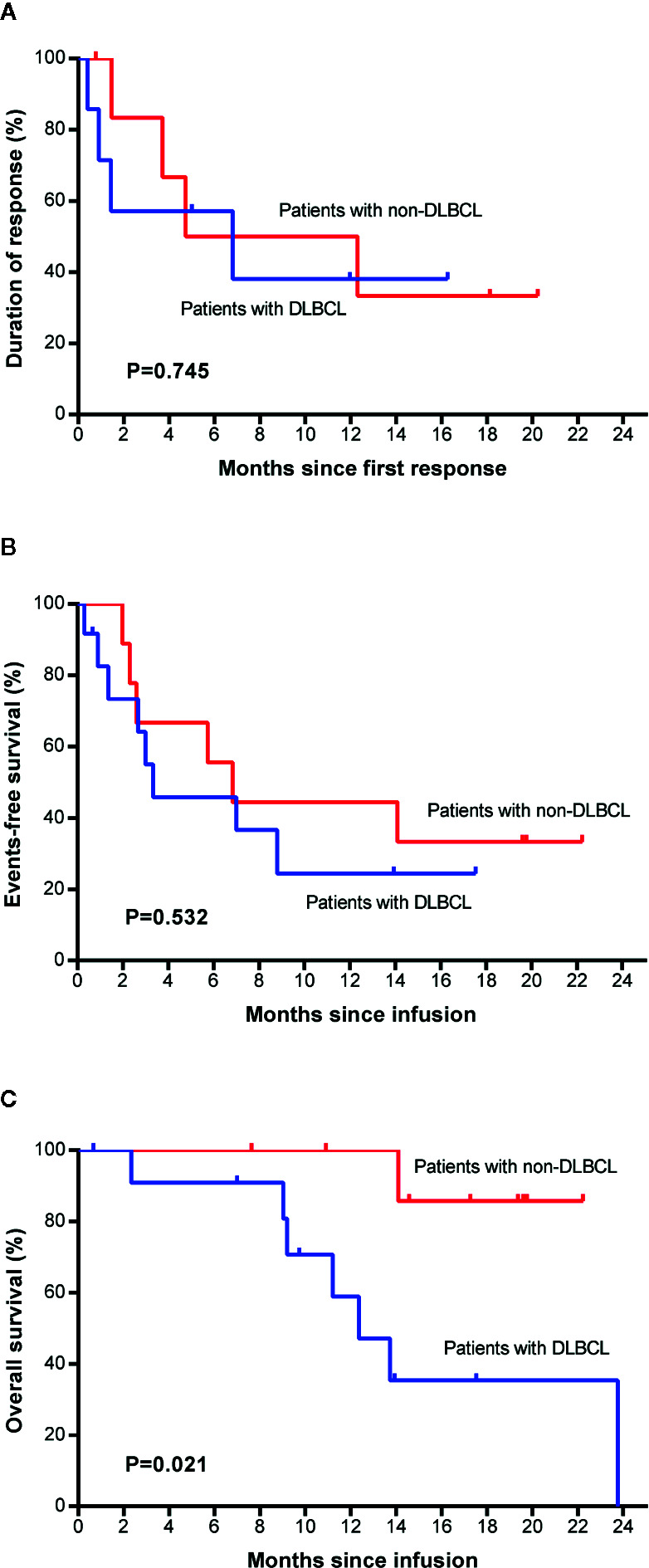
Survival comparison between patients with DLBCL and with non-DLBCL. Duration of response **(A)**, event-free survival **(B)**, and overall survival **(C)** between the two groups. Non-DLBCL, patients with non-Hodgkin’s lymphomas other than DLBCL. DLBCL, diffuse large B cell lymphoma.

No significant differences were found between patients with DLBCL and patients with non-DLBCL regarding DoR (P=0.745, **Figure 5A**) or EFS (P=0.532, **Figure 5B**), but the non-DLBCL group outperformed the DLBCL group in OS (P=0.021, HR=0.116, **Figure 5C**), which was mentioned in the section about the univariate analysis. The median OS of the non-DLBCL group has not been reach, while it was 12.4 months (95% CI, 8.9 to 15.9 months) in the DLBCL group. Eighty-six percent (95% CI, 33 to 98) of patients in the non-DLBCL group are estimated to survive after 22 months, while 35% (95% CI, 9 to 64) of patients in the DLBCL group are estimated to survive after 22 months.

### CAR-T Cell Expansion

**Figure 6:**
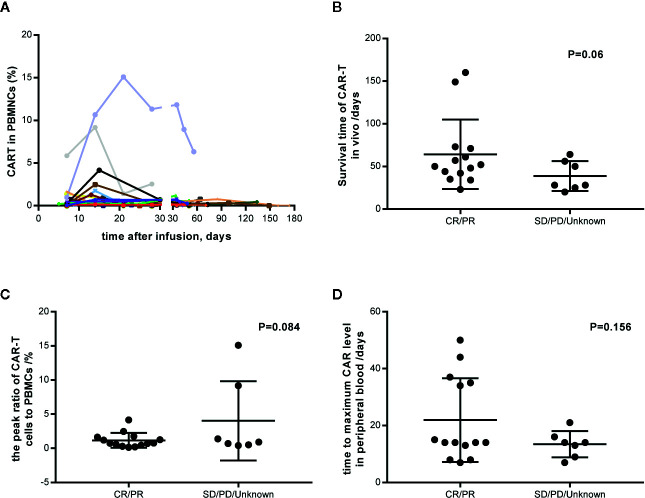
CAR-T cell expansion *in vivo* and its relation with response. **(A)** Individual concentration-time profile in all patients. **(B)** Survival time of CAR-T cells *in vivo* in responders and nonresponders. **(C)** The peak ratio of CAR-T cells to PBMCs *in vivo* in responders and nonresponders. **(D)** The time to maximum CAR level in peripheral blood. PBMCs, peripheral blood mononuclear cells. CR, complete response; PR, partial response; SD, stable disease; PD, progressive disease.

4SCAR19 T cell expansion was monitored after infusion on a regular schedule. Until the data cutoff date, all the expansion data were collected and varied among the patients (**Figure 6A**). CAR-T cells were still detectable *in vivo* 160 days after infusion in a patient with partial response. The median survival time of CAR-T cells in patients with response was longer than that in patients without response (51 *versus* 28 days); however, there was no significant difference between the two groups (P=0.06, **Figure 6B**), which may be related to the small sample size. Similar results were observed in the peak of the ratio of CAR-T cells to mononuclear cells (0.74 *versus* 0.9%, P=0.084, **Figure 6C**) and the time to maximal CAR level in peripheral blood (14 *versus* 14 days, P=0.156, **Figure 6D**) in patients with a response *versus* in those without a response, which indicated that CAR-T cell expansion *in vivo* had little effect on clinical outcome. There was no significant difference in CAR-T cell dose and clinical response (P=0.332), and no correlation between CAR-T cell dose and amplification peak was observed (P=0.339, data not shown). Seventy-six percent (16/21) of patients maintained a complete response or partial response after CAR-T cells became undetectable.

### Safety

In our study, diverse adverse events were observed in all patients receiving 4SCAR19 T cell infusion. The most common adverse events of any grade were reduced white blood cell count (in 100% of patients), decreased neutrophil count (100%), anemia (90%), decreased platelet count (62%), and infection (33%) (**Table 3**). The most common adverse events of grade 3 or 4 included decreased neutrophil count (in 76% of patients), decreased white blood cell count (71%), decreased platelet count (29%), anemia (24%), febrile neutropenia (19%), and pulmonary infection (10%). After symptomatic treatment, the patient’s symptoms were alleviated. There were no deaths due to infusion of CAR-T cells.

**Table 3:** Adverse events of interest in the treatment.

	Any, n (%)*****	Grade 1****	Grade 2****	Grade 3****	Grade 4****	Grade 5****
**Any**	21 (100%)					
**White blood cell count decreased**	21 (100%)	2 (10%)	4 (19%)	9 (43%)	6 (29%)	0
**Neutrophil count decreased**	21 (100%)	1 (5%)	4 (19%)	4 (19%)	12 (57%)	0
**Anemia**	19 (90%)	6 (29%)	8 (38%)	5 (24%)	0	0
**Platelet count decreased**	13 (62%)	4 (19%)	3 (14%)	3 (14%)	3 (14%)	0
**Hypogammaglobulinemia^†^**	9 (43%)	—	—	—	—	—
**Infection**	7 (33%)	0	4 (19%)	3 (14%)	0	0
**Upper respiratory tract infection**	4 (19%)	0	4 (19%)	0	0	0
**Pulmonary infection**	2 (10%)	0	0	2 (10%)	0	0
**Urinary tract infection**	1 (5%)	0	0	1 (5%)	0	0
**Cutaneous infection**	1 (5%)	0	1 (5%)	0	0	0
**Increased AST**	6 (29%)	5 (24%)	1 (5%)	0	0	0
**Pyrexia**	5 (24%)	4 (19%)	1 (5%)	0	0	0
**Febrile neutropenia**	4 (19%)	0	0	4 (19%)	0	0
**CRS**	3 (14%)	3 (14%)	0	0	0	0
**CRES**	1 (5%)	0	0	1 (5%)	0	0
**Pancytopenia last for ＞28 days**	1 (5%)	—	—	—	—	—

*All the data are shown as n (%).

^†^Hypogammaglobulinemia and pancytopenia are not included in CTCAE V4.03, so their severity was not graded.

— Not applicable.

AST, aspartate aminotransferase; CRS, cytokine release syndrome; CRES, CAR-T-cell-related encephalopathy syndrome.

Cytokine release syndrome (CRS) was observed in 3 (14%) patients on days 2, 6, and 7, respectively, accompanied by increased serum levels of C-reactive protein (CRP) and interleukin-6 (IL-6), and lasted for 2 to 10 days. All cases were grade 1, with symptoms of pyrexia, fatigue, diarrhea, etc. Patient symptoms were ameliorated after antipyretic therapy, empirical antibiotic treatment, and supporting therapy, and serum levels of CRP and IL-6 also gradually decreased. None of the patients received tocilizumab or glucocorticoids. Unfortunately, none of these three patients achieved remission (two had stable disease, and one had disease progression).

One patient developed grade 3 CAR-T cell-related encephalopathy syndrome (CRES) on day 33 after infusion, with clinical manifestations of cognitive impairment, aphonia, unresponsiveness, mild irritability and headache, febrile neutropenia, hypogammaglobulinemia (decreased by 0.71-fold), and upper respiratory tract infection, but not accompanied by abnormal elevations of CRP or IL-6. The patient did not have central nervous system diseases before CAR-T cell infusion. After dehydration and intravenous dexamethasone treatment, the CRES was resolved. The patient had a poor prognosis, with disease progression occurring 29 days after the partial response.

In this study, both CRS and CRES occurred during the proliferation phase of CAR-T cells, suggesting that their occurrence may be related to the activation and proliferation of CAR-T cells *in vivo* (**Supplemental Figure S1**). There was a significant difference in the peak serum concentration of CRP (P=0.032), pretreatment LDH (P=0.02), and CAR-T cell infusion dose (P=0.039) between patients with and without CRS/CRES (**Supplemental Figure S2**). However, no significant difference was found in the peak serum concentration of IL-6 (P=0.107) or the peak of CAR-T cell expansion *in vivo* (P=0.654) between the two groups.

Cytopenia was the most common adverse event in this study, and all patients experienced a reduction in at least one line of cellular elements in the circulating blood. Sixty-two percent of patients received recombinant human granulocyte colony stimulating factor (rhG-CSF), and three patients were given platelet and/or erythrocyte transfusion as supplementation. Some patients experienced prolonged cytopenia for more than 28 days or 3 months (**Supplemental Table S3**), and one patient had pancytopenia lasting longer than 28 days.

According to the detection of lymphocyte subsets after CAR-T cell infusion, all 19 patients tested experienced B cell dysplasia, which lasted for a maximum of 251 days. Nine of these patients (47%) developed hypogammaglobulinemia from 2 to 35 days after CAR-T cell infusion, with a minimum reduction of 0.57 times (relative to the lower limit of normal), and the hypogammaglobulinemia could be cured after intravenous injection of human immunoglobulin. Upper respiratory tract infection occurred in three of these nine patients. These results show that B cell dysplasia can be controlled clinically, and patients can well tolerate the long-term loss of B cells.

## Discussion

There has been great development of CAR-T cell therapies in treating both hematological neoplasms and other neoplasms in recent years, and these therapies show better efficacy in CD19-positive lymphomas than in CD19-negative lymphomas, with a promising ORR of 52~83% (6–8). In this study, 67% of patients had an objective response, which was consistent with previously reported results. The results of this trial and our previous trial prove the effectiveness and potential of our 4th-generation CAR-T cells (18). In addition, CD27, a costimulatory molecule, is not inferior to 4-1BB, OX40 and ICOS. Similar to 4-1BB and OX40, CD27 also belongs to the tumor necrosis factor (TNF) receptor family related to TNF receptor-associated factor (TRAF) and plays a role in the generation of T cell memory as a costimulatory molecule (24). CD27 costimulation has been proven to enhance the survival, expansion, and antitumor functions of CAR-T cells *in vitro* and *in vivo* (25, 26). This was the first clinical application of CAR-T cells containing the CD27 intracellular domain, and the results were encouraging.

Previous studies have paid little attention to non-DLBCL patients, and our study included patients with MCL, FL, mucosa-associated lymphoid tissue (MALT), and Burkitt lymphoma (BL), as well as DLBCL, PMBCL, and high-grade B cell lymphoma. The results were encouraging in that 78% of non-DLBCL patients achieved an objective response. However, more cases with non-DLBCL need to be included for further research to confirm whether non-DLBCL patients benefit more than DLBCL patients from the 4SCAR19 T cell product. The outcome of DLBCL patients performed badly in this trial, which may be attributed to individual differences, CART dose, tumor burden, and sample size. The subgroup factors, such as age, sex, disease status, and extranodal lesions, did not affect the response rate, which is consistent with previous studies. Interestingly, we found that IPI score, disease subtype, and remission status after CAR-T cell infusion were related to patient survival time. In the era of chemotherapy, the IPI score is widely used to assess the prognosis of NHL patients. Whether IPI is applicable in the era of cellular immunotherapy is unknown. Our research suggests that the IPI score is related to the OS of patients after CAR-T cell therapy. We speculate that patients with high IPI scores have a poor prognosis because these patients have a lower response to lymphodepleting therapy than patients with low IPI scores, which has a substantial negative effect on subsequent CAR-T cell therapy. Most of the efficacy results of the 4SCAR19 T cell are in line with the results of the registrational trials of the second generation CAR, and that it is because the bone of CAR structure was similar that both of them consist of scFv, costimulatory domains, and CD3zeta.

It is universally acknowledged that the most common and severe adverse events related to CAR-T cell therapy are CRS and neurologic toxicity (22, 23). In our study, these two adverse events were less frequent and less severe than they were in studies of axicabtagene ciloleucel and tisagenlecleucel treatment of B cell lymphomas (6–8, 27). Compared with other studies, the rate of CRS of any grade (14%) observed in our study was lower than that in other studies, which ranged from 58 to 93%, and there was no grade 3 or 4 CRS. Moreover, only one patient developed neurological symptoms. Of note, none of the patients who received 4SCAR19 T cell infusion were given glucocorticoids (except for the patient with CRES) or tocilizumab within 1 month after infusion. The low incidence of CRS/CRES in our research may result from three factors: 1) the unique 4SCAR design and a shortened *ex vivo* incubation time; 2) lower CAR-T cell infusion dose; and 3) administration of bridging chemotherapy for patients with high tumor burden. Of note that we have observed a low cytokine release profile and a slower kinetic for the 4^th^ generation CAR than for the 2^nd^ and 3^rd^ generation CARs (18, 21). In addition, it has been reported that patients who have a high tumor burden and receive a high dose of CAR-T cells are more likely to develop CRS/CRES through intensive activation of CAR-T cells and cytokine secretion.

Cytopenias were the most frequent adverse events in this trial. Eighty-six percent of patients experienced cytopenias after lymphodepleting therapy, and the cell numbers increased after CAR-T cell infusion with or without growth factor supplementation, indicating that lymphodepleting therapy was responsible for the cytopenias. The frequency of patients with grade 3 or 4 cytopenias (neutropenia, anemia, and thrombocytopenia) at any time (76, 24, and 29%, respectively) was lower than that in JULIET (81, 58, and 54%) and ZUMA-1 (93, 66, and 58%) (7, 8). However, the number of patients with prolonged cytopenias beyond 28 days or 3 months in this study was similar to those in JULIET and ZUMA-1 (28). Since prolonged cytopenias are common after lymphodepleting treatment and CAR-T cell infusion, it is essential to monitor patient blood cell counts regularly and administer rhG-CSF or blood transfusion if needed.

In contrast to second generation CAR, the fourth generation CAR has a potential advantages of superior safety profile that the frequencies or severity of adverse events were much lower. Besides, the strategy of suicide switch allow the researchers to eliminate supernormally activated CART cells. Further research on the mechanism behind the safety of fourth-generation CAR-T cell therapy is needed. Another reason for the low incidence of CRS may be related to the tumor type, as patients with solid tumors seem to have a lower incidence than patients with hematological tumors. For example, the rate of CRS of any grade (77 *versus* 58%) and of grade 3/4 (46 *versus* 22%) in patients with B cell lymphoblastic leukemia was higher than that in patients with lymphoma (8, 29). Similar results were observed in other tumor types, such as neuroblastoma, in which no CRS was reported after administration of anti-GD2 CAR-T cells (30).

Since severe toxicities may contribute to a poor effect on efficacy and survival, efforts have been made to improve the safety of CAR-T CAR T cell therapy. The JCAR017 construct, which contains a truncated, cell surface version of human epidermal growth factor (EGFRt) and can be recognized and disabled by cetuximab, showed a superior safety profile in TRANSCEND NHL 001; the frequency of CRS (all, 35%; grade 3 or 4, 1%) and neurotoxicities (all, 19%; grade 3 or 4, 12%) were lower than those in previous reports (31, 32). Of note, only a single patient experienced severe CRS in this trial. Similar results were observed in CALM, which featured treatment with UCAR-T19, an allogeneic product containing an RQR8 domain that allows removal of UCAR-T19 by rituximab (33). These results and our experience indicated the potential of fourth-generation CAR-T cell products containing “safety switches” in decreasing CAR-T cell-related toxicities.

In summary, these results indicated that patients with relapsed or refractory B cell non-Hodgkin’s lymphoma who received 4SCAR19 T cell therapy had high levels of durable response and low levels of adverse events. The IPI model is suitable for evaluating the prognosis of patients receiving CAR-T cell therapy.

## Data Availability Statement

All datasets presented in this study are included in the article/**Supplementary Material**.

## Ethics Statement

The studies involving human participants were reviewed and approved by Medical Ethics Committee of Zhujiang Hospital of Southern Medical University. The patients/participants provided their written informed consent to participate in this study.

## Author Contributions

XZ, ST and CW wrote the manuscript draft. YHL, ST, and RH designed the study and managed the patients. XZ, LD, CS, YH, ZL, and WZ contributed to patient management. CY and JY participated in the registration of the clinical research. AW, ML, and ZG were responsible for clinical trial recruitment. CJ and YCL produced CAR-T cells, and LC designed the CAR-T cells structure and guide CAR-T cell production. CW collected the data and conducted the analysis. ST, YQL, and YHL revised the manuscript. All authors contributed to the article and approved the submitted version.

## Funding

This work was supported by the Science and Technology Program of Guangzhou, China (grant number 201704020216), the Frontier Research Program of Guangzhou Regenerative Medicine and Health Guangdong Laboratory (grant number 2018GZR110105014), the Clinical Research Startup Program of Southern Medical University by High-Level University Construction Funding of Guangdong Provincial Department of Education [grant number LC2016ZD027], the Natural Science Foundation of Guangdong Province, China [grant number 2018B030311042], the Science and Technology Planning Project of Guangdong Province, China (grant number 2017A020215043), the Science and Technology Planning Project of Guangdong Province, China (grant number 2017A020215183), and the research funds from Science and Technology Planning Technical Research Project of Shenzhen (JCYJ20170817172416991 and JCYJ20170817172541842).

## Conflict of Interest

The authors declare that the research was conducted in the absence of any commercial or financial relationships that could be construed as a potential conflict of interest.
