# Supplementary material for: Phase I Trial of Fourth-Generation Anti-CD19 Chimeric Antigen Receptor T Cells Against Relapsed or Refractory B Cell Non-Hodgkin Lymphomas
Source: Front Immunol. 2020 Nov 27;11:564099. doi: 10.3389/fimmu.2020.564099 (PMC7731732; doi:10.3389/fimmu.2020.564099)
Supplement: Supplementary file 1 [file DataSheet_1.pdf]

Supplementary Material

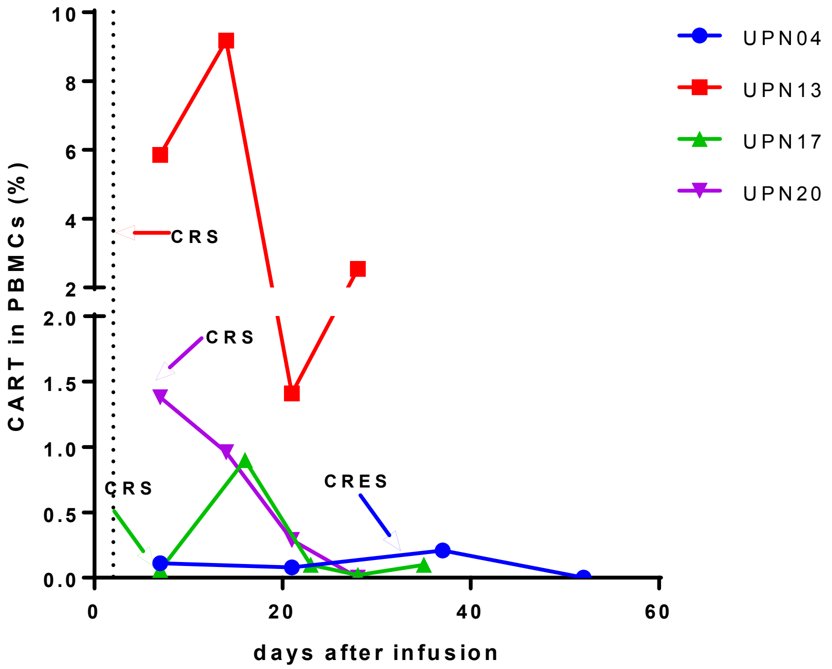

Supplementary Figure 1. Relationship between the occurrence of CRS/CRES and expansion of CART cells in vivo.

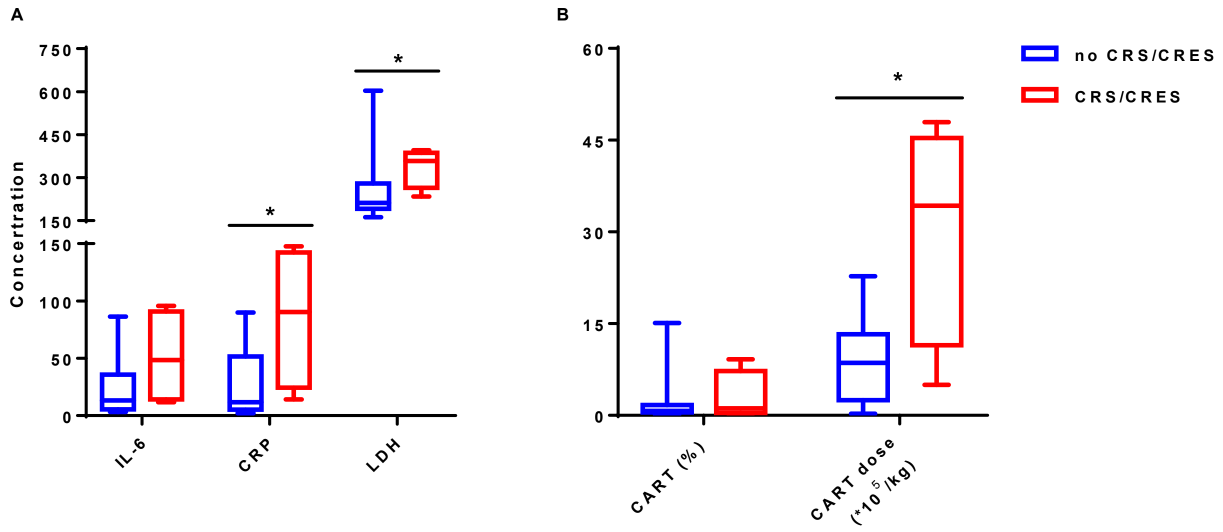

Supplementary Figure 2. Analysis of risk factors related to the occurrence of CRS/CRES. The relationship between the occurrence of CRS/CRES and the peak serum concentration of IL-6 and CRP, the concentration of LDH before infusion (**A**), the infusion dose of CART cells, and the highest level of CART cell expansion in vivo (**B**).

Supplementary Table 1. 4SCAR19 T cell dose administered.

	**Patients (N=21)**
CAR-positive viable T-cell dose infused (10^5 cells/kg)	
Mean (SD)	13.08 (12.89)
Median	8.87
Range	0.28~47.96
Total cell dose infused (10^6 cells/kg)	
Mean (SD)	2.75 (1.48)
Median	2.47
Range	0.39~7.44

Supplementary Table 2. Differences in remission time after infusion in patients with complete remission and partial remission.

**Efficacy**	**Time post-infusion/days**		**Statistic**	**Sig.**
**CR**	Mean		49.78	0.509
	95% CI for Mean	Lower Bound	41.31	
		Upper Bound	58.25	
	Median		54	
	Std. Deviation		11.020	
	Minimum		34	
	Maximum		63	
**PR**	Mean		55.00	
	95% CI for Mean	Lower Bound	36.90	
		Upper Bound	73.10	
	Median		61	
	Std. Deviation		14.577	
	Minimum		29	
	Maximum		63	

Supplementary Table 3. Prolonged cytopenias in all patients in the study.

**Prolonged Cytopenias, %**	**Patients (N=21)**		
	at any time	at ≥ 28 days	at ≥ 3 months
**Neutropenia ≥ grade 3**	76%	29%	14%
**Anemia ≥ grade 3**	24%	14%	0%
**Thrombocytopenia ≥ grade 3**	29%	29%	10%
